# Anatomical Remembrancer, or Complete Pocket Anatomist

**Published:** 1871-11

**Authors:** 


*The Anatomical Remembrancer, or Complete Pocket Anatomist.* By C. E. Isaacs, M.D. New York: William Wood & Co., 1871.

This little work contains a concise description of the structures of the human body. It has passed its third edition, with such corrections and additions by the author, as are most essential to the medical student in his perusal of practical anatomy. As a guide in the teachings of the lecture room, it is unequalled. The book has a very neat appearance, and we recommend it to the student as a very useful companion.

